# Characterization of the Mode of Action of Aurodox, a Type III Secretion System Inhibitor from Streptomyces goldiniensis

**DOI:** 10.1128/IAI.00595-18

**Published:** 2019-01-24

**Authors:** Rebecca E. McHugh, Nicky O’Boyle, James P. R. Connolly, Paul A. Hoskisson, Andrew J. Roe

**Affiliations:** a Institute of Infection, Immunity and Inflammation, University of Glasgow, Glasgow, United Kingdom; b Strathclyde Institute of Pharmacy and Biomedical Sciences, University of Strathclyde, Glasgow, United Kingdom; University of California, Davis

**Keywords:** bacteria, *Escherichia*, infection, inhibitor, secretion, virulence

## Abstract

Recent work has demonstrated that the polyketide natural product Aurodox from Streptomyces goldiniensis is able to block the pathogenesis of the murine pathogen Citrobacter rodentium. In this work, we aimed to gain a better understanding of the mechanism of action of the compound. We show that Aurodox downregulates the expression of the type III secretion systems of enteropathogenic and enterohemorrhagic Escherichia coli. Furthermore, we have used transcriptomic analysis to show that Aurodox inhibits the expression at the transcriptional level by repressing the master regulator, *ler*. Our data support a model in which Aurodox acts upstream of *ler* and not directly on the secretion system itself. Finally, we have shown that Aurodox, unlike some traditional antibiotics, does not induce expression of RecA, which is essential for the production of Shiga toxin. We propose that these properties nominate Aurodox as a promising antivirulence therapy for the treatment of these infections.

## Abstract (precis)

Recent work has demonstrated that the polyketide natural product Aurodox from Streptomyces goldiniensis is able to block the pathogenesis of the murine pathogen Citrobacter rodentium. In this work, we aimed to gain a better understanding of the mechanism of action of the compound.

## INTRODUCTION

Enterohemorrhagic Escherichia coli (EHEC) is a zoonotic pathogen responsible for foodborne outbreaks of diarrhea which can escalate to hemorrhagic colitis and hemolytic-uremic syndrome (HUS) (1). Symptoms of enteropathogenic Escherichia coli (EPEC) infection are typically less severe; however, this pathotype remains the leading cause of diarrheal deaths in the developing world (2). As with many other Gram-negative pathogens, EHEC and EPEC utilize the type III secretion system (T3SS) to promote host colonization (3). The T3SS is a needle-like apparatus which facilitates the translocation of effector proteins to the epithelial cells of the gut. Through the injection of these proteins, which include the translocated intimin receptor (Tir) and the mitochondrion-associated protein (Map), EHEC and EPEC are able to polymerize actin filaments and form intimate junctions with epithelial cells, where several other virulence factors are expressed to destabilize cellular processes (4–6). In the case of EHEC infection, symptoms can extend beyond the intestine, as strains typically carry phage-derived Shiga toxins (Stx) which target organs such as the kidneys and brain (7). This often leads to the life-threatening condition HUS (1).

The T3SS of EHEC and EPEC is encoded by the highly conserved locus of enterocyte effacement (LEE) island (8). This pathogenicity island contains 42 genes on five conserved operons and is regulated by the master regulator, Ler. In turn, *ler* is regulated by specific regulators, such as GrlA and GrlR, in addition to global regulators which mediate LEE expression in response to environmental stimuli (9).

The reliance of EHEC and EPEC on the T3SS to initiate infection has identified it as a target for novel therapies to fight infection. Typically, these are part of a wider antivirulence approach in which the aim is to prevent infection by the inhibition of a single virulence factor without inducing a reduction in growth (10). Currently, treatment of EHEC infections with traditional antibiotics is not recommended due to stimulation of the bacterial SOS response (11). In response to DNA damage caused by antibiotics, the SOS response protein RecA is overexpressed, which results in activation of the Stx-encoding phage (12). Hence, Stx production is upregulated and symptom severity increases. Additionally, the disruption to the native gut microbiome by broad-spectrum antibiotics can have negative consequences for the patient (13). As EHEC and EPEC infections are typically cleared naturally, antivirulence approaches to treatment of EHEC and EPEC represent an exciting new strategy for the treatment of these infections. In addition, compounds that do not affect bacterial growth or survival reduce the evolutionary selective pressure on strains resistant to the treatment (10), enhancing the long-term viability of the therapy.

Small-compound inhibitors of the EPEC and EHEC T3SS have previously been identified (14, 15). Notably, members of the salicylidene acylhydrazide (SA) family have been shown to inhibit T3S in a range of enteric pathogens, including EPEC, EHEC, and Salmonella enterica (16). However, these compounds were found to bind to several bacterial protein targets, and their mode of action has been shown to result from synergistic effects arising from a perturbation of the function of several conserved metabolic proteins (17). Therefore, the conclusion was that although effective, the SAs were rather promiscuous (17).

Several antivirulence compounds are actually natural products of other bacterial species (16). Aurodox, a specialized metabolite of Streptomyces goldiniensis, was shown by Kimura et al. (18) to inhibit the translocation of T3SS encoded effectors in EPEC, without affecting growth *in vitro*. Low concentrations (1.5 µM) were shown to inhibit secretion, and abolition of detectable effector proteins was observed at 6 µM. Moreover, the effect of the compound *in vivo* was tested through the use of a Citrobacter rodentium murine infection model in which it was shown that mice treated with the compound survived lethal infections with limited effects on the intestinal tract. Although the effects of the compound on T3S in EPEC were characterized, the wider effects and the mechanism of action of the compound were not elucidated (18). Therefore, there is a need to gain a better understanding of the mechanism of action of Aurodox.

Aurodox was originally discovered in 1973 as an antibiotic compound with antibacterial effects upon Gram-positive pathogens such as Streptococcus pyogenes and Staphylococcus aureus (19). Aurodox has since been well characterized in terms of its bactericidal mechanism, with a mild effect upon E. coli growth reported using concentrations greater than 1 mg/ml, 200 times higher than used in T3S assays (5 µg/ml) (20). Vogeley et al. determined the crystal structure of Aurodox bound to elongation factor Tu (EF-Tu) of Thermus thermophilus, which confirmed their hypothesis that Aurodox initiated a conformational change in EF-Tu which prevented it from dissociating from the ribosome, blocking elongation activity (21). The limited spectrum of antibiotic activity of Aurodox has resulted in its being rejected as an antibiotic to treat infections in humans, yet the discovery of the novel T3SS inhibitory properties suggests that Aurodox may be a candidate drug for repurposing.

In this study, we characterized the effect of Aurodox on T3S in EHEC O157:H7 (TUV93-0), EPEC O127:H6 (E2348/69), and Citrobacter rodentium (ICC168), demonstrating that the Aurodox effects are independent of growth. Furthermore, we used transcriptomic analysis to show that Aurodox inhibits the T3SS at the level of transcription by repression of the LEE master regulator, *ler*. Furthermore, we suggest a model in which Aurodox acts upstream of Ler and not directly on the T3SS itself. Finally, we show that Aurodox does not induce expression of RecA, which is essential for the production of Stx. We propose that these properties make Aurodox a candidate antivirulence therapy for the treatment of EPEC and EHEC infections.

## RESULTS

### Aurodox inhibits the translocation of T3SS-associated effector proteins without affecting growth.

In a previous study, Kimura et al. (18) demonstrated that Aurodox reduced the translocation of EPEC effector proteins in a concentration-dependent manner. We aimed to explore the effect of the compound on other LEE-encoding pathogens, primarily EHEC (TUV93-0) and C. rodentium (ICC168), and determine whether the mechanism of T3SS inhibition was independent of an inhibition in growth.

Each strain was cultured in media appropriate for the expression of the T3SS (22). Aurodox was added to the cultures at the point of inoculation at increasing concentrations ranging from 1.5 µg/ml to 5 µg/ml (1.2 to 6 µM), and bacteria were grown through 4 generations to an optical density at 600 nm (OD_600_) of 0.7 to 0.9. Supernatant proteins were precipitated and whole-cell lysates prepared as a comparator. The fractions were separated by SDS-PAGE and stained with Coomassie brilliant blue.

**FIG 1:**
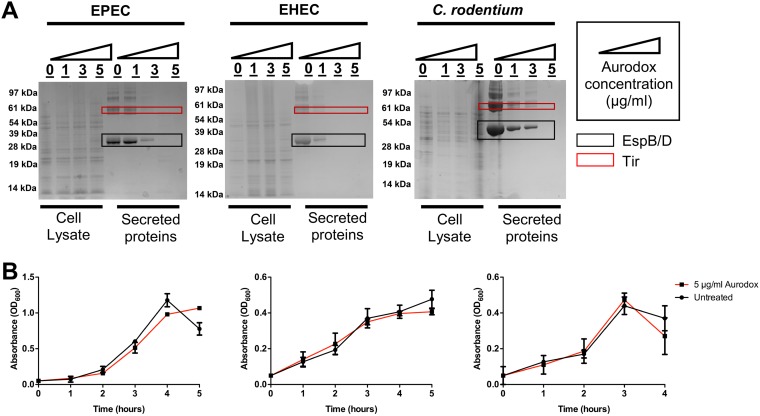
Aurodox inhibits secretion of T3SS-associated effector proteins in EPEC, EHEC, and Citrobacter rodentium without affecting bacterial growth. (A) Secreted protein fractions were prepared by culturing strains in T3S-inducing medium and precipitated from the supernatant using trichloroacetic acid. Whole-cell fractions were prepared by lysis of cell pellets. Samples were resolved using SDS-gel electrophoresis followed by Coomassie staining. Marked protein bands were excised and trypsin digested for mass spectrometry identification of proteins. (B) Aurodox does not inhibit the growth of EPEC, EHEC, or C. rodentium in T3S-inducing medium. EHEC was grown in MEM-HEPES, and EPEC and C. rodentium were grown in DMEM. Growth rates were determined spectrophotometrically (OD_600_; *n* = 3). Error bars plotted are SDs. Changes in growth were not statistically significant.

For the supernatant fractions, a concentration-dependent reduction in T3SS-associated effector proteins was observed (Fig. 1A). The dominant bands from the gel were excised to permit in-gel trypsin digestion and analysis by tandem mass spectrometry. This confirmed that for all three pathogens, the two most dominant bands were comprised of three well-known effector proteins: Tir, EspB, and EspD (Fig. 1A and Tables S3 to S5 in the supplemental material). In contrast, there was no change in the profile of the cellular proteins (Fig. 1A), indicating that the mechanism was not due to a generic block in protein synthesis. Furthermore, these data allowed us to rule out an effect on general secretion as EspP, which is secreted in a Sec-dependent manner, was consistently detected in high abundance, even after Aurodox treatment (Table S4).

At the highest concentration used (5 µg/ml), Aurodox did not inhibit growth of EPEC, EHEC, or C. rodentium (Fig. 1B). Importantly, no statistically significant decrease in cell viability was observed (data not shown). Therefore, at the concentration of Aurodox used, the mechanism of T3S inhibition is independent of any defect in growth or viability. This result confirms data by Kimura et al. (18) showing no effect of Aurodox on growth of EPEC and extends our understanding to the related pathogens C. rodentium and EHEC.

### Aurodox inhibits the ability of EHEC to attach to and efface epithelial cells.

Previous studies using a murine infection model tested the inhibitory effects of Aurodox on C. rodentium pathogenicity, demonstrating a marked improvement in the survival of infected mice and a reduction in colon damage when treated with Aurodox (18); however, the effects were not demonstrated at the cellular level.

**FIG 2:**
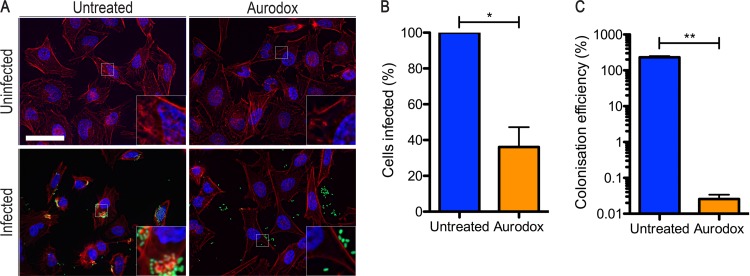
Effect of Aurodox on EHEC infection of epithelial cells and A/E lesion formation. (A) Representative microscopy images from EHEC cell infection assay. Cells were infected with 10^7^ EHEC cells transformed with p*rpsM-gfp* (green) to facilitate quantification and imaging. HeLa cells were actin stained with phalloidin-Alexa Fluor 555 (red) and mounted in Vectashield with DAPI (blue). Scale bar represents 50 μm. Insets contain a ×4 magnification of the indicated area. (B) Colonization was quantitated by counting the numbers of cells possessing EHEC on their surface and expressing as a percentage of the total. (C) Following infection, HeLa cells were washed to remove nonadherent bacteria and subsequently lysed to release colonized bacteria. The CFU of EHEC in the lysate were enumerated, and colonization efficiency was calculated by expressing as a percentage of the inoculum. Significance was calculated by paired Student’s *t* test. *, *P* < 0.05; **, *P* < 0.01.

To investigate the effects of Aurodox on the attachment of EHEC to host cells, which is known to be driven via the T3SS, we used an *in vitro* infection assay. The effect of Aurodox (5 µg/ml) on uninfected HeLa cells was tested to confirm that there was no overt cytotoxicity of the compound (Fig. 2A). HeLa cells were infected with 10^7^ EHEC bacteria constitutively expressing green fluorescent protein (GFP). Host cell actin cytoskeleon was stained with phalloidin-Alexa Fluor 555. In addition, cell lysis was carried out to quantify the attached bacteria as a percentage of the initial inoculum. Epifluorescence microscopy images were used to quantify the infected cells and to investigate any effects on cell morphology.

At a mutliplicity of infection of 150, untreated EHEC produced consistent infections, with all HeLa cells showing adherent bacteria. The HeLa cells displayed morphological changes associated with bacterial infection, including actin condensation caused by lesion formation and cell rounding (Fig. 2A). Addition of Aurodox substantially reduced both phenotypes, with only 36% of cells becoming infected and a marked reduction in attaching and effacing (A/E) lesions. Indeed, the infections by EHEC were typically restricted to a small number of bacteria per cell (<5). Quantification of bacterial adherence efficiency by CFU counts showed a >3-log reduction in EHEC colonisation when treated with Aurodox (Fig. 2C), demonstrating the potent antivirulence capacity of Aurodox.

### Aurodox inhibits expression of multiple virulence genes, including the LEE.

**FIG 3:**
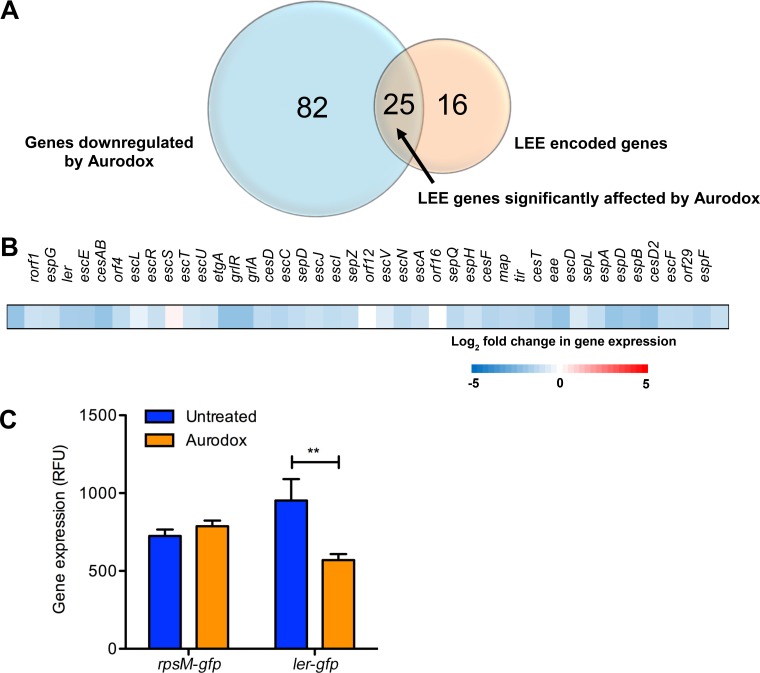
Transcriptional changes induced by Aurodox in EHEC. (A) Venn diagram representing overlap between genes significantly downregulated by Aurodox (>1.5 fold; *P* < 0.05) and LEE genes. (B) Heat map representation of log_2_ fold change in gene expression after treatment with Aurodox. (C) Effect of Aurodox on expression of *ler* and the housekeeping gene *rpsM*. Aurodox was added at 5 µg/ml and expression measured after 4 h of growth. Expression of *rpsM* was not significantly altered in contrast to that of Ler (*P* = 0.009). Error bars plotted are equivalent to the SDs (*n* = 3). **, *P* < 0.005.

To gain insights into the possible molecular mechanism underlying the inhibition of the T3SS, we used whole-transcriptome analysis. EHEC was grown in minimal essential medium (MEM)-HEPES with or without 5 mg/ml of Aurodox, and RNA was extracted from triplicate cultures. Transcripts were mapped to the EHEC EDL933 reference genome, and the mean fold change was calculated. In total, 84 chromosomal genes and 4 pO157 genes were significantly downregulated (using a fold change of −1.5 and an EDGE test *P* value of <0.05) (Fig. 3A and Table S6). Consistent with the secretion data, analysis of locus of enterocyte effacement (LEE) transcription revealed downregulation of the island after treatment with Aurodox (Fig. 3B), with 25 of the 41 LEE genes significantly downregulated, including the master regulators *ler* and *grlA* (9) (Fig. 3A and B). Aurodox also repressed expression of genes encoding non-LEE-encoded effector proteins secreted by the T3SS, such as NleB and EspG. Moreover, there was no significant downregulation of genes encoding T3SS-independent effector proteins, such as EspP.

The data also revealed that many genes in the colanic acid biosynthesis operon were downregulated in the presence of Aurodox. Our initial results show that 20 of the 21 genes in this operon are downregulated in response to Aurodox, with 15 of the 20 genes showing significant changes (*P* = 0.05) (Table S6). Notably, expression of *rcsA*, a key regulator of colanic acid biosynthesis that is known to form part of the Ler-dependent regulon (23), was downregulated 2.5-fold. These data suggest that suppression of the colanic acid operon is likely due to expression changes in the key regulators RcsA and Ler. These changes could indicate further potential of Aurodox for the treatment of EHEC infections, as colanic acid production is required *in vivo* during infection (24).

In addition, 103 genes were upregulated in the presence of Aurodox, which include various metabolic genes (*prpR*, *trpA*, and *trpB*), and genes encoding sensory proteins (e.g., *narQ*). Importantly, there was no upregulation of SOS response-associated genes or virulence genes. The most upregulated gene was *ecpD*, encoding a putative fimbrial chaperone protein, with a log_2_ fold change of 5.

### Aurodox inhibits T3SS expression through downregulation of the master regulator Ler.

To confirm the virulence-related Aurodox targets identified by RNA sequencing (RNA-seq), we used GFP gene reporter assays to validate observed changes in gene expression. GFP reporter plasmids for *ler* and the housekeeping gene *rpsM* were introduced into strains, and fluorescence was measured during exponential phase. This showed that in EHEC expression of *ler* was significantly reduced in the presence of Aurodox (Fig. 3C). There was no significant change in expression of the housekeeping gene *rpsM* (Fig. 3C), consistent with the RNA-seq data. A reduction in *ler* expression was also observed for EPEC (Fig. S1), implying a common mechanism by which Aurodox causes a reduction of T3SS expression.

### Overexpression of Ler overrides EHEC inhibition of the T3SS by Aurodox.

**FIG 4:**
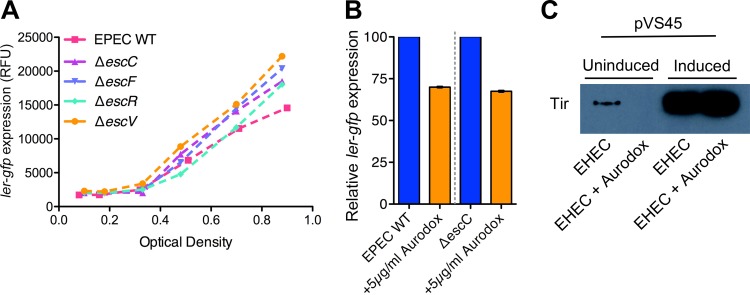
Transcriptional repression of *ler* does not occur as a result of the interaction of Aurodox with the T3S needle. (A) Analysis of *ler* expression in EPEC mutants with deletions of genes encoding structural components of the T3SS. Mutant strains were transformed with p*ler-gfp*. Optical density and fluorescence were measured at hourly intervals and *ler* expression was quantified as relative fluorescence units (fluorescence/OD_600_). (B) Relative *ler* expression during exponential phase in WT EPEC versus EPEC Δ*escC* treated with Aurodox. Expression of *ler* in treated samples is expressed as a percentage of untreated samples. (C) Effect of Ler overexpression on the Aurodox phenotype. Arabinose was added to induce expression, and the effect of Aurodox on Tir expression was determined by immunoblotting.

Inhibition of the T3SS by Aurodox could be explained by a number of mechanisms. These include (i) directly inhibiting Ler expression or function, (ii) binding to a component of the T3SS resulting in negative feedback of *ler*, or (iii) affecting an upstream regulator of *ler*. To test if expression of structural components of the T3SS affected expression of Ler, we used a series of EPEC deletion mutants in various apparatus proteins. The rationale was that if a structural component was a target of Aurodox, then this would have to result in downregulation of *ler* and therefore the other genes in the T3SS and the wider Ler regulon. However, analysis of four structural components of the T3SS apparatus proteins showed that there was no effect on *ler* transcription when assayed using a GFP reporter (Fig. 4A). Moreover, addition of Aurodox resulted in reduced expression of *ler* in both wild-type (WT) EHEC and an *escC* deletion mutant (Fig. 4B). These data show that there is no apparent feedback from structural components on the expression of the master regulator of the T3SS itself and that Aurodox results in downregulation of *ler*, irrespective whether the T3SS is fully assembled.

We then questioned if a regulator upstream or downstream of Ler was the target. To address this, EHEC was transformed with the pVS45 plasmid carrying *ler* under transcriptional control of an arabinose-inducible promoter (25). By selectively overproducing Ler we could test the sensitivity of EHEC to Aurodox-mediated T3SS inhibition. The transformed strain was grown in media to induce LEE expression both in the presence and absence of 5 µg/ml of Aurodox and 2% arabinose to induce expression of Ler. The fractions were analyzed using immunoblotting with antibodies against the T3SS protein Tir. In the absence of arabinose (uninduced Ler), the addition of Aurodox results in T3SS inhibition as seen in previous experiments (Fig. 4C). However, overexpression of Ler by addition of arabinose completely overcame the phenotype of T3SS-mediated repression normally associated with addition of Aurodox (Fig. 4C). This result indicates that Aurodox acts either by directly acting on Ler or by a mechanism involving an upstream regulator of Ler.

### Aurodox does not induce the SOS response in EHEC.

**FIG 5:**
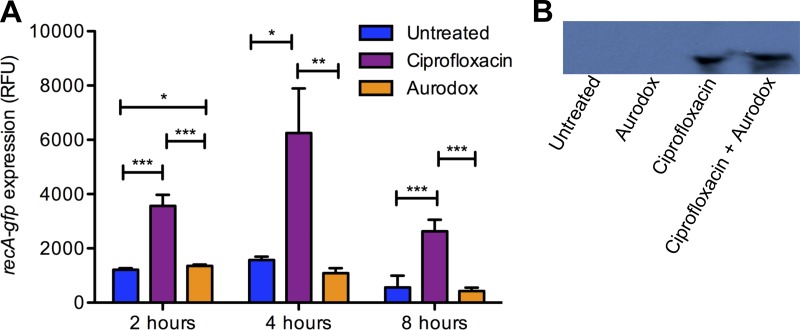
Analysis of the effect of Aurodox on RecA-mediated Stx expression. (A) Analysis of *in vitro recA-gfp* expression in Aurodox-treated EHEC. Aurodox and ciprofloxacin were added at 6 µM. Error bars correspond to SDs (*n* = 4). *P* values for Aurodox-treated versus ciprofloxacin-treated bacteria were <0.01. *, *P* < 0.05; **, *P* < 0.005; ***, *P* < 0.001. (B) Immunoblot analysis of Stx expression in untreated, Aurodox-treated, ciprofloxacin-treated, and Aurodox- and ciprofloxacin-treated C. rodentium DBS100. Primary antibodies for Shiga toxin B subunit (6 kDa) were used.

Antimicrobial compounds that induce DNA damage are known to stimulate the bacterial SOS response. A key protein in this regulon is RecA, a coprotease that functions in the autocatalytic cleavage of the LexA and the λ repressors, resulting in derepression of approximately 40 genes involved in the SOS response and, importantly, an increased expression of bacteriophage-encoded Stx (26). As a potential treatment for EHEC infection, we therefore aimed to investigate if Aurodox had any undesirable effects on Stx expression. RNA-seq data did not reveal differential expression of SOS regulated genes (as defined by Fernández et al. [27]) in response to Aurodox. To validate this, EHEC was transformed with a plasmid containing a *recA-gfp* fusion and gene expression was measured over a time course (Fig. 5). Ciprofloxacin was used a positive control of RecA expression and provided log-fold stimulation compared with the uninduced control (Fig. 5). In contrast, the data show that Aurodox does not induce *recA* transcription in EHEC at the 4- and 8-h time points (Fig. 5). The data imply that addition of Aurodox does not induce the SOS response and therefore, by implication, Stx expression, enhancing the potential of the compound to be used as a treatment for EHEC infections.

To test this hypothesis, we directly examined the effect of Aurodox on Stx expression *in vitro*. To test this, Citrobacter rodentium DBS100 was used. This strain has been lysogenized by an Stx2dact-producing phage and produces functional Stx. This strain was cultured in the presence of Aurodox, ciprofloxacin, and both Aurodox and ciprofloxacin. The secreted proteins were harvested and probed using an antibody for the beta subunit of the Shiga toxin (Fig. 5B). The resulting bands showed that Aurodox does not induce the expression of Stx, in contrast to Ciprofloxacin. These findings therefore enhance the potential of the compound to be used as a treatment for EHEC infections.

## DISCUSSION

The central role of T3SSs in the pathogenesis of many bacterial species provides the impetus to develop strategies that interfere with their function. To this end, “virulence blockers” have been described that selectively target the expression or function of T3SSs in a number of bacterial species, including Yersinia pseudotuberculosis, Salmonella enterica serovar Typhimurium, and Chlamydia pneumoniae (28, 29). While screening programs can readily find compounds that inhibit the T3SS, identifying inhibitors that work both *in vitro* and *in vivo* with high selectivity has proven challenging. Therefore, the discovery by Kimura et al. (18) that Aurodox could protect mice from a lethal infection with C. rodentium was an important one.

In this study, we attempted to address three questions. First, can Aurodox block the function of the T3SS in EHEC O157:H7, as well as EPEC and C. rodentium? This is important to address because there are very limited treatment options for EHEC infections due to complications associated with Shiga toxin expression. Second, are there inhibitor-specific transcriptional responses that may help us understand the mode of action? Third, does Aurodox alter the bacterial the SOS response affecting expression of bacteriophage-encoded Stx?

In agreement with Kimura et al. (18), we observed that Aurodox resulted in a concentration-dependent inhibition of T3S in EPEC and C. rodentium. The same result was found in EHEC, a result that shows a likely common mechanism of inhibition. Importantly, at the concentrations used, there were no effects on bacterial growth rate or viability in any of the species tested. This finding suggests that the mechanism by which Aurodox inhibits the T3SS is distinct from the reported target of bactericidal activity, elongation factor Tu (EF-Tu) (21), a vital component of the protein biosynthesis machinery. It is notable that Aurodox is an extremely poor antibiotic against E. coli, with previous work showing limited inhibition zones at 1 mg/ml, some 200× the concentrations used to inhibit the T3SS (19). Furthermore, the distinct natures of the mechanisms of T3S inhibition and bactericidal activity have positive implications for resistance. Although resistance to Aurodox as an antibiotic may arise through mutations in EF-Tu, these mutations would not confer resistance to Aurodox as an inhibitor of the T3SS or restore virulence. Although it is possible mutations in *ler* or its regulators may result in resistance to Aurodox as a T3S inhibitor, these would not be strongly selected for in the same way as conventional antibiotics.

Our transcriptomic data provide the first insight into how Aurodox affects global gene expression. Remarkably, only 3.24% of the genome showed a significant up- or downshift in transcription. This shows excellent specificity and selectivity of inhibition, both very desirable qualities for an antivirulence compound (10). Perhaps predictably, much of the LEE was seen to be downregulated, including transcription of the master regulators *ler* and *grlA* (9). Further, many of the genes affected were part of the Ler regulon, suggesting a mode of action centered around this protein. Critically, overexpression of Ler by an inducible expression system, completely overrides the effects of Aurodox on secretion of Tir via the T3SS. Our data also confirm that deletion of components of the T3SS system, such as structural proteins, does not lead to transcriptional feedback on *LEE1*. In conjunction with the transcriptomics data, our working model is that Aurodox binds a target that affects transcription of *ler*. The complexity and high molecular weight of Aurodox suggest that the compound may have the ability to compromise the integrity of the outer membrane on cell entry and hence activate the membrane stress response sigma factor, RpoE. Increased expression of this sigma factor has been reported to reduce T3S via downregulation of *ler*, particularly in the presence of zinc (30). However, our transcriptomal analysis has shown a 1.54-fold downregulation of the sigma factor, and therefore, we believe that this pathway is unlinked. Elucidating specific targets for antivirulence compounds is often challenging (31), and proving a specific target for Aurodox would be problematic because it is a complex natural product, making labeling or modification extremely difficult.

We have also demonstrated, for the first time, that Aurodox does not stimulate expression of RecA, a key protein that helps mediate the SOS response in E. coli (32). The SOS response is known to be a key regulator of lambdoid bacteriophage transcription by stimulating autocleavage of the phage repressor, cI, in response to DNA damage, triggering a cascade that leads to transcription of the Q antiterminator transcript and the lytic cycle (33). As both the *stxA* and *stxB* genes in EHEC strains are located on the genomes of resident prophages (34), it is not surprising that they are upregulated by the SOS response, which can induced by treatments that damage bacterial DNA, including UV light, selected antibiotics, and mitomycin C. This response to traditional broad-spectrum antibiotics has made treatment of EHEC problematic and highlights an important advantage of Aurodox: it can suppress virulence without the “sting in the tail” associated with Shiga toxin expression and damage.

A further interesting aspect of Aurodox biology would be to explore any wider effects on the microbiota. The gut microbiota has long been recognized to contribute to health and disease by influencing gut maturation, host nutrition, and pathogen resistance (35). Moreover, the density and diversity of the gut microbiota offers ample opportunities for the horizontal transfer of genetic material, including antibiotic resistance genes. Therefore, it would be important to understand if Aurodox was indeed highly targeted toward a small subset of pathogens or if it had any undesirable effects on the wider gut microbiota, which could be monitored using methods such as metagenomics. Overall, our work shows that Aurodox works in a highly targeted manner that supports its strong consideration for repurposing as treatment against EHEC infection in humans.

## MATERIALS AND METHODS

### Bacterial strains, plasmids, and growth conditions.

The strains used in this study are detailed in Table S1 in the supplemental material. The plasmids used in this study are described in Table S2. To induce T3SS expression, EHEC TUV93-0 was grown in minimal essential medium (MEM)-HEPES (Sigma, St. Louis, MO) with 5.62 g/liter of l-glutamine and EPEC was grown in Glutamax-Dulbecco modified Eagle medium (DMEM) (Sigma) at 37°C at 180 rpm. C. rodentium was also grown in Glutamax-DMEM (Sigma) and was cultured statically at 37°C with 5% CO_2_. Growth and cell viability assays were carried out in lysogeny broth. For selection of plasmids, chloramphenicol or ampicillin was included at 20 or 50 μg/ml, respectively.

### Acquisition and storage of Aurodox.

Aurodox was purchased in its pure form (Enzo Life Sciences, Farmingdale, NY). A 1-mg/ml stock solution was prepared by dissolving the compound in dimethyl sulfoxide (DMSO). The overall concentration of DMSO was <0.5% in all experiments. We also purified Aurodox directly from *S. goldiniensis* according to the method of Berger et al. (19) and found the same effects. However, for consistency we used commercially available Aurodox.

### Analysis of secreted proteins.

Overnight cultures were used to inoculate each strain into the appropriate prewarmed medium to an initial OD_600_ of 0.05. The strains were grown with increasing concentrations of Aurodox to an OD_600_ of 0.7 to 0.9 before being centrifuged at 3,800 rpm for 10 min to separate the cell mass. Cell lysates were prepared using BugBuster protein extraction buffer (Merck, NJ). The supernatant was removed, and proteins were precipitated by the addition of trichloroacetic acid to a concentration of 10% and incubated overnight at 4°C. The suspension was centrifuged at 3,800 × *g* for 1 h to form a protein pellet which was suspended in 100 µl of Tris-HCl (pH 8.0). The samples were then mixed 1:1 with loading dye (Novex, Waltham, MA) and run on a 4 to 12% SDS-PAGE gel at 120 V for 1 h. Gels were subsequently stained with Coomassie brilliant blue stain (Novex) and destained in water overnight. When required, bands were excised for subsequent in-gel digestion and analysis. Proteins analyzed by tandem mass spectrometry were given a MASCOT score to indicate the probability of the identification being correct.

### Analysis of the effect of Aurodox on *in vitro* growth and cell viability.

Overnight cultures of EHEC (TUV93-0), EPEC (E2348/69), and C. rodentium (ICC186) were used to inoculate 200-ml Erlenmeyer flasks containing 50 ml of LB broth with or without 5 µg/ml of Aurodox to an initial OD_600_ of 0.05. This was carried out in triplicate. At each time point, 100 µl of culture was removed and diluted 1/10 in LB medium, and the OD_600_ was measured by spectrophotometry. The standard error mean of optical densities was calculated, and a standard curve was plotted using GraphPad Prism. The lines were fitted according to Weilbul formula and error bars were plotted as standard deviations from the means. *P* values were determined using an unpaired *t* test. For cell viability assays, each strain was grown to and OD_600_ of 3.0 both with and without Aurodox, and CFU were determined through the serial dilution method.

### Infection of HeLa epithelial cells with EHEC O157:H7.

A 24-well tissue culture plate containing coverslips was seeded with 10^7^ HeLa cells in MEM-HEPES containing 5.62 g/liter of l-glutamine and 10% fetal calf serum. These were left to grow overnight. In parallel, two cultures of TUV93-0 p*rpsM-gfp* (with or without 5 µg/ml of Aurodox) were grown in 5-ml volumes under T3SS-inducing conditions until an OD_600_ of 0.6 was reached. The HeLa cells were washed once with phosphate-buffered saline (PBS) before 500 µl of fresh MEM-HEPES (with or without 5 µg/ml of Aurodox) was added, in addition to 15 µl of bacterial culture. The plate was then centrifuged at 250 × *g* for 3 min and was left to incubate initially for 1 h. Serial dilutions of the inoculum were carried out and spotted onto LB agar to determine the CFU (as described above). After the initial hour, cells were washed three times with sterile PBS, and 600 µl of fresh MEM-HEPES with or without 5 µg/ml of Aurodox was added before incubation for a further 3 h. To quantify the adherent EHEC bacteria on treated and untreated cells, the cells were washed four times with sterile PBS and lysed through the addition of 1% Triton in PBS and incubation at room temperature for 10 min. Serial dilutions were spotted onto LB agar to determine the adherent CFU. Colonization efficiency was calculated as a percentage of the initial inoculum. To visualize the infections using wide-field epifluorescent microscopy, cells on coverslips were fixed with 4% paraformaldehyde (20 min at room temperature) before permeabilization with 0.1% Triton in PBS (5 min at room temperature) and staining with phalloidin-Alexa Fluor 555 (Thermo Fisher; 1 in 500 dilution and 1 h at room temperature). Cells were mounted using Vectashield with 4′,6-diamidino-2-phenylindole (DAPI) and sealed with clear nail polish. Images were acquired at a magnification of ×400 on a Zeiss Axioimager M1. For representative images, 11 z-slices of 0.55 μm were acquired before deconvolution using default settings in Zeiss Zen Pro software. Host cells were classified as infected if any bacteria were seen to be associated with them.

### mRNA extraction.

EHEC (TUV93-0) was cultured as described above in triplicate, both with and without 5 µg/ml of Aurodox. The cultures were mixed with 2 volumes of RNAprotect reagent (Qiagen, Valencia, CA) at room temperature before being centrifuged at 3,800 × *g* to harvest a cell pellet. RNeasy kit (Qiagen) was used to extract total RNA before TURBO DNase (Ambion, Carlsbad, CA) was used to remove genomic DNA. Furthermore, a MICROBExpress mRNA enrichment kit (Ambion) was used to enrich samples for mRNA. The quality of the mRNA was determined using an Agilent 2100 bioanalyzer at the University of Glasgow Polyomics Facility.

### Transcriptome analysis using RNA sequencing.

cDNA synthesis and sequencing were performed at the University of Glasgow Polyomics Facility (Illumina NextSeq 500), obtaining 75- or 100-bp single-end reads. Treated and untreated samples were sequenced in triplicate. FastQC (Babraham Bioinformatics, Cambridge, UK) was used to for quality control. Reads were trimmed accordingly using CLC Genomics Workbench (CLC Bio, Aarhus, Denmark). Trimmed reads were mapped to the EDL933 reference genome (NCBI accession number NC_002655.2), allowing for 3 mismatches per read and at least 5 reads per feature. Analysis of differential expression was performed using the Empirical analysis of DGE tool, which implements the EdgeR Bioconductor tool. Differentially expressed genes were identified using a positive or negative absolute fold change of ≥1.5 and a corrected *P* value of ≤0.05 (false-discovery rate of 5%). Gene Ontology functional grouping was summarized according to information available on Colibase and the RegulonDB. Figures were generated using GraphPad Prism and Microsoft Excel.

### *In vitro* GFP fusion reporter assays.

Electrocompetent EHEC, EPEC, and C. rodentium cells were transformed with the promoter-*gfp* reporter plasmids listed in Table S2. The transformants were inoculated in to 10 ml of the appropriate medium and cultured as previously stated. Samples were removed at the desired time points to take measurements of the OD_600_. To determine the overall fluorescence, 200 µl of culture was transferred into a black 96-well plate and fluorescence was read with excitation of 485 nm and emission at 550 nm, using the FLUOstar Optima fluorescence plate reader system (BMG Labtech, UK). This was carried out in triplicate. To determine the normalized fluorescence value, the background fluorescence intensity from untransformed cells was first subtracted and the subsequent values were normalized by dividing fluorescence by OD_600._ The data are the means for the three samples, and error bars represent the standard deviations.

### Overexpression of *ler*.

Electrocompetent EHEC cells were transformed with pVS45 plasmid containing the *ler* gene under the control of an arabinose-induced promoter and grown on 50 µg/ml of ampicillin. A transformant colony was then used to prepare an overnight culture, which was subsequently inoculated into T3SS-inducing conditions as described above. At an OD_600_ of 0.2, 2% arabinose was added. The supernatant protein fractions were then precipitated and analyzed using SDS-PAGE as previously explained. Proteins for Western blot analysis were run on a 4 to 12% bis-Tris gel and transferred to an Amersham ECL nitrocellulose membrane (GE Healthcare, Chicago, IL) using the Nupage Novex gel transfer system (Invitrogen, Carlsbad, CA). Blocking was then carried out using 5% skimmed milk powder in PBS-Tween (PBST). The membrane was then incubated with the anti-Tir primary antibody overnight in 1% milk powder–PBST buffer at a 1:1,000 dilution. Antibodies for GroEL (Abcam) were used as a control for bacterial cell lysis at the same dilution. The following morning, the membrane was washed three times with PBST for 10 min before being incubated for 1 h with anti-mouse horseradish peroxidase (HRP)-conjugated secondary antibody at a 1:2,000 dilution in 1% milk in PBST. The membrane was again washed three times with PBST. Finally, the membrane was developed with SuperSignal West Pico chemiluminescent ECL (Thermo Fisher) substrate for 5 min before being transferred to a dark room in a cassette and exposed to X-ray film for 30 s.

### Detection of Shiga toxin expression in C. rodentium DBS100 by Western blotting.

C. rodentium DBS100 containing the Stx phage was cultured in 2 ml of DMEM at 37°C and 200 rpm until an OD_600_ of 0.6 was reached. The whole-cell fraction was removed from by centrifugation at 3,800 × *g* for 10 min. Secreted proteins were harvested as previously described. Proteins for Western blot analysis were run on a 4 to 12% bis-Tris gel and transferred to an Amersham ECL polyvinylidene difluoride (PVDF) membrane (GE Healthcare, Chicago, IL) using the Nupage Novex gel transfer system (Invitrogen, Carlsbad, CA). No blocking was carried out. The membrane was incubated with anti-Stx (beta subunit) antibody (1/1,000 concentration) overnight at 4°C and washed three times with PBST. The membrane was incubated for 1 h with anti-mouse HRP-conjugated secondary antibody at a 1:2,000 dilution in 1% milk in PBST and washed three times with PBST. Finally, the membrane was developed with SuperSignal West Pico chemiluminescent ECL (Thermo Fisher) substrate for 5 min before being transferred to a dark room in a cassette and exposed to X-ray film for 30 min.

### Accession number(s).

The sequence reads in this paper have been deposited in the European Nucleotide Archive under study PRJEB29967.

## Supplementary Material

Supplemental file 1
