# Supplementary material for: Characterization of the Mode of Action of Aurodox, a Type III Secretion System Inhibitor from Streptomyces goldiniensis
Source: Infect Immun. 2019 Jan 24;87(2):e00595-18. doi: 10.1128/IAI.00595-18 (PMC6346137; doi:10.1128/IAI.00595-18)
Supplement: Supplemental file 1 [file 117c39064df564581bb917f8d4a3cbab_IAI.00595-18-s0001.pdf]

Table S1: Strains used in this study

Strain	Description	Source
**TUV 93-0**	Shiga-toxin negative variant of EHEC O157:H7, derived from EDL933 outbreak strain	(1)
**E2348/69**	Wild type EPEC O127:H6	(2)
**Δ*escC***	Δ*escC* derivative of EPEC E2348/69	(2)
**Δ*escF***	Δ*escF* derivative of EPEC E2348/69	(2)
**Δ*escR***	Δ*escR* derivative of EPEC E2348/69	(2)
**Δ*escV***	Δ*escV* derivative of EPEC E2348/69	(2)
**ICC168**	Wild type *Citrobacter rodentium*	(2)
**DBS100**	DBS100 lysogenized with Φ 1720a02 ΔRz::cat Δstx2dactAB::kan, CmR, KmR	(3)

Table S2: Plasmids used in this study

Plasmid	Description	Source
pAJR70	Empty plasmid vector, Chloramphenicol resistance marker.	(4)
p*ler-gfp*	*ler* promoter fused to gfp. Chloramphenicol resistance marker.	(4)
p*rpsM-gfp*	*rpsM* promoter fused to gfp. Chloramphenicol resistance marker.	(4)
pVS45	Plasmid encoding *ler* with arabinose inducible promoter. Ampicillin resistance marker	(5)
p*recA-gfp*	*recA* fused to gfp. Chloramphenicol resistance marker.	(6)
p*tir*-gfp	*tir* fused to gfp. Chloramphenicol resistance marker	

Table S3: MASCOT results for EPEC secreted protein bands

Protein	Mass	MASCOT score	Matches
**EspB**	31564	498	9/9
**EspD**	39033	1204	21/21
**Tir**	57954	2116	43/43

Table S4: MASCOT results for EHEC secreted protein bands

Protein	Mass	MASCOT score	Matches
**EspB**	31564	648	11/11
**EspD**	39033	874	14/14
**Tir**	57954	2336	50/50
**EspP**	135881	2655	47/47

Table S5: MASCOT result for *C. rodentium* secreted protein bands

Protein	Mass	MASCOT score	Matches
**EspB**	31564	-	-
**EspD**	39033	-	-
**Tir**	57954	377	6/6

Table S6: Chromosomal EHEC TUV93-0 genes downregulated in the presence of Aurodox.

Gene	Fold Change	P Value
espH	-1.51	0.04
metE	-1.51	0.01
ydgA	-1.52	0.00827
Z2386	-1.53	0.0021
escJ	-1.53	0.03
rpoE	-1.54	0.000591
Z2387	-1.55	0.00607
adhE	-1.56	0.000144
yeaD	-1.6	0.000375
sepZ	-1.6	0.0019
espF	-1.61	0.00231
yhcK	-1.61	0.05
spfA	-1.61	0.00155
xasA	-1.63	0.04
sepL	-1.63	0.00117
rrsE	-1.63	0.01
ptsG	-1.66	0.000000407
escI	-1.66	0.01
mokP	-1.67	0.01
abgT	-1.68	0.04
escN	-1.69	0.0000174
cesD	-1.7	0.000775
espJ	-1.71	0.000591
rrsD	-1.71	0.006
sepQ	-1.72	0.0000902
escF	-1.72	0.00782
rrsB	-1.74	0.00216
map	-1.74	0.000582
tktB	-1.76	0.00121
lifA	-1.77	0.03
tir	-1.78	0.00000692
osmC	-1.79	0.0000687
escC	-1.8	0.0000001
espX	-1.81	0.00149
pyrI	-1.82	0.00268
cesF	-1.82	0.00867
ysgA	-1.83	0.000126
poxB	-1.84	0.04
espL2	-1.87	0.00881
nleB	-1.89	0.02
orf29	-1.89	0.000106
rrfF	-1.93	0.00827

katE	-1.93	0.04
espW	-1.99	4.08E-09
rrfG	-2	0.00206
cesT	-2.02	4.63E-08
Z2063	-2.02	0.0000147
escE	-2.03	0.000533
Z5138	-2.04	0.0000436
yjcO	-2.13	0.00135
cesD2	-2.13	1.01E-11
manZ	-2.16	8.56E-13
espD	-2.18	5.84E-08
nleA	-2.2	0.0000411
manY	-2.21	1.69E-14
manX	-2.29	4.59E-19
espA	-2.32	4.26E-10
eae	-2.34	0
rorf1	-2.34	0.02
espB	-2.36	5.06E-10
fruA	-2.44	0.00000123
grlA	-2.45	0.00000008
rcsA	-2.47	1.67E-08
rrlG	-2.53	0.00527
yneB	-2.54	0.0047
ssrS	-2.57	0.000106
cpsB	-2.74	0.00827
ffs	-2.8	0.03
wzc	-2.97	0.03
fruB	-3.07	0.0000147
yghX	-3.51	0.00607
fruK	-3.59	0.000538
wcaH	-3.8	0.04
wcaG	-3.83	0.000000108
yjbE	-3.93	0.04
wcaD	-3.94	0.000563
wcaE	-3.98	0.00704
gmd	-5.04	5.22E-16
yebV	-5.89	0.02
wcaI	-6.07	1.64E-08
wcaC	-10.12	0.01
cpsG	-12.12	0.00000259
yjbF	-43.7	0.00554

Table S7: Genes upregulated in the presence of Aurodox

Gene	Fold Change	P value
yheN	1.51	0.00000166
Z2005	1.52	0.00000151
yafH	1.53	0.00000191
pyrF	1.53	0.00000211
yqiB	1.53	0.00000142
trmA	1.57	0.00000173
yegQ	1.58	0.00000195
Z4370	1.58	0.00000284
sapA	1.59	0.00000169
hemA	1.59	0.00000443
yfhJ	1.6	0.00000159
folX	1.6	0.00000145
gabP	1.61	0.0000134
yfcI	1.61	0.00000143
mdaA	1.61	0.0000014
yliG	1.61	0.00000227
stpA	1.62	0.00000146
yhiW	1.62	0.00000157
ycaD	1.62	0.00000115
bcr	1.63	0.00000205
terC	1.63	0.00000125
ydeA	1.65	0.00000243
yafS	1.67	0.00000161
yccJ	1.67	0.00000161
abc	1.67	0.00000269
hypF	1.68	0.00000127
pheA	1.69	0.00000154
ygaF	1.69	0.0000126
yfbS	1.7	0.00000188
yfhE	1.71	0.0000069
leuC	1.71	0.00000764
pheP	1.72	0.0000026
yedV	1.72	0.00000183
leuB	1.73	0.00000588
ycgN	1.73	0.00000112
ydcP	1.75	0.00000323
fadB	1.75	0.00000154
nrdD	1.76	0.000000974
ygaT	1.77	0.0000152
sodB	1.78	0.00000114
fimB	1.78	0.00000164
trpC	1.78	0.00000712
bigA	1.8	0.00000249
prpE	1.81	0.00000134

yieP	1.81	0.00000165
leuD	1.81	0.00000392
Z2806	1.82	0.0000014
yfcD	1.83	0.00000279
yciA	1.84	0.00000161
narQ	1.85	0.00000125
acrD	1.85	0.00000109
ubiX	1.85	0.00000217
leuA	1.89	0.00000923
Z4832	1.91	0.00000209
Z2491	1.96	0.0000022
espR	1.99	0.000000894
yjhS	2	0.00000103
ycdZ	2.02	0.00000163
ycfS	2.03	0.00000321
Z4833	2.11	0.00000233
yadB	2.12	0.00000143
yhfC	2.18	0.000005
trpB	2.25	0.0000161
trpA	2.27	0.0000104
phoE	2.37	0.000000808
Z0701	2.39	0.000000646
ykgF	2.4	0.000000647
frdB	2.43	0.000000755
metF	2.59	0.00000174
yjcZ	2.63	0.000000653
torR	2.65	0.000000871
ebgR	2.9	0.00000124
yjiY	2.96	0.00000328
aldH	3	0.0000013
yafO	3	0.00000055
yliF	3	0.00000055
ymdF	3.14	0.000000991
hcaR	3.2	0.000000882
Z3235	3.22	0.000000609
Z1644	3.27	0.00000105
yfhH	3.29	0.000000774
bssR	3.39	0.00000172
mtr	3.66	0.0000141
CII	4.01	0.000000447
narJ	4.19	0.000000679
ybiH	4.93	0.00000108
trpE_1	5.23	0.00000223
dicB	5.62	0.000000395
prpR	5.62	0.000000395
Z2986	5.63	0.000000397
trpD	6.05	0.00000744
hycA	6.27	0.000000452
yaeJ	6.28	0.00000111
Z4398	6.28	0.000000452

yjgZ	8.26	0.00000062
napC	8.97	0.000000682
ydfZ	9.64	0.000000739
torD	10.09	0.0000025
torA	13.4	0.0000104
sorA	16.27	0.000000344
torC	18.24	0.00000365
Z6041	18.81	0.000000401
cmtA	18.84	0.000000402
ecpD	33.8	0.000000739

Figure S1: Effect of Aurodox on *ler* expression in EPEC.

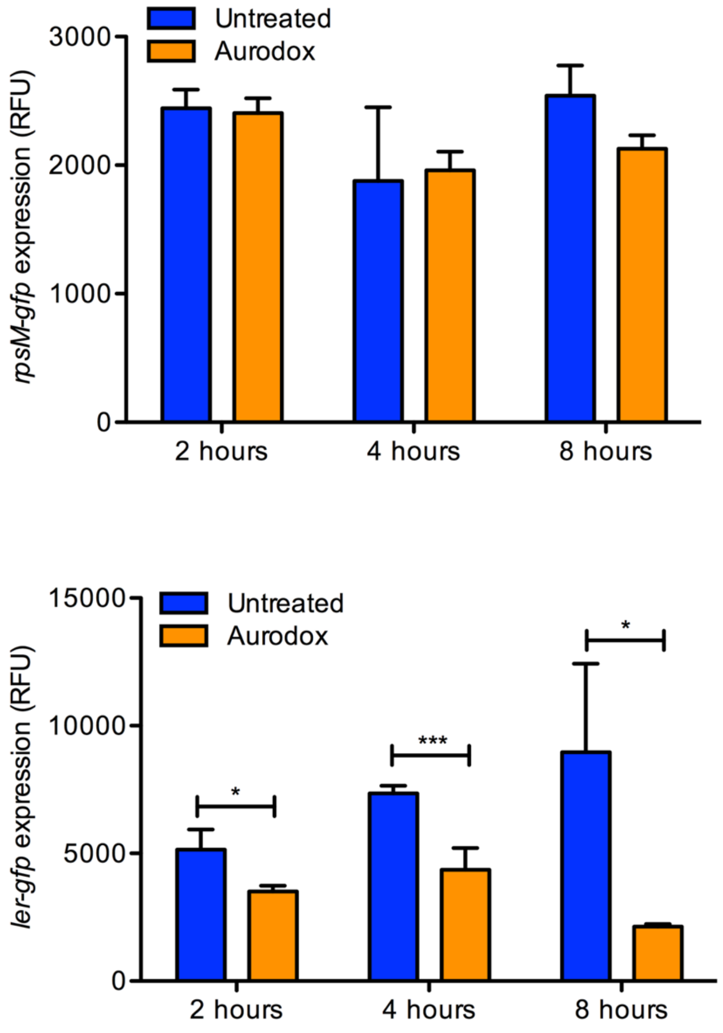

Figure S1: Effect of Aurodox on expression of *ler* compared to housekeeping gene *rpsM* in EPEC. EPEC transformed with p*ler-gfp*/p*rpsM-gfp* was cultured in the presence or absence of 6 μM Aurodox as indicated, n = 3. Error bars plotted are equivalent to the standard deviation from the mean. Normalised relative fluorescence values (RFU) were calculated by dividing the raw fluorescence value by the Optical Density (600nm). No changes in RpsM expression were statistically significant. Reduction in *ler* expression at all time points was statistically significant (P = 0.02, 0.004 and 0.025 respectively).

Supplementary References

1. Campellone KG, Robbins D, Leong JM. 2004. EspFU is a translocated EHEC effector that interacts with Tir and N-WASP and promotes Nck-independent actin assembly. Dev Cell 7:217–228.

2. Deng W, Puente JL, Gruenheid S, Li Y, Vallance BA, Vázquez A, Barba J, Ibarra JA, O'Donnell P, Metalnikov P, Ashman K, Lee S, Goode D, Pawson T, Finlay BB. 2004. Dissecting virulence: Systematic and functional analyses of a pathogenicity island. Proc Natl Acad Sci U S A 101:3597–3602.

3. Mallick EM, McBee ME, Vanguri VK, Melton-Celsa AR, Schlieper K, Karalius BJ, O'Brien AD, Butterton JR, Leong JM, Schauer DB. 2012. A novel murine infection model for Shiga toxin-producing Escherichia coli. J Clin Invest 122:4012–4024.

4. J. RA, W. NS, J. SK, M. YH, A. DT, Matthew O, J. MI, Megan P, J. WM, E. SDG, L. GD. 2004. Co-ordinate single-cell expression of LEE4- and LEE5-encoded proteins of *Escherichia coli* O157:H7. Mol Microbiol 54:337–352.

5. Vanessa S, L. MJ, M. DR, Gad F, Adam CJ, William N, B. KJ. 2002. Activation of enteropathogenic *Escherichia coli* (EPEC) LEE2 and LEE3 operons by Ler. Mol Microbiol 38:781–793.

6. Zaslaver A, Bren A, Ronen M, Itzkovitz S, Kikoin I, Shavit S, Liebermeister W, Surette MG, Alon U. 2006. A comprehensive library of fluorescent transcriptional reporters for Escherichia coli. Nat Methods 3:623–628.
